# Fully Automated Lipid Pool Detection Using Near Infrared Spectroscopy

**DOI:** 10.1155/2016/1487859

**Published:** 2016-08-17

**Authors:** Elżbieta Pociask, Joanna Jaworek-Korjakowska, Krzysztof Piotr Malinowski, Tomasz Roleder, Wojciech Wojakowski

**Affiliations:** ^1^Department of Automatics and Biomedical Engineering, AGH University of Science and Technology, Aleja Mickiewicza 30, 30-059 Krakow, Poland; ^2^Faculty of Health Science, Institute of Public Health, Jagiellonian University Medical College, 31-531 Krakow, Poland; ^3^Third Department of Cardiology, Medical University of Silesia, 40-635 Katowice, Poland

## Abstract

*Background*. Detecting and identifying vulnerable plaque, which is prone to rupture, is still a challenge for cardiologist. Such lipid core-containing plaque is still not identifiable by everyday angiography, thus triggering the need to develop a new tool where NIRS-IVUS can visualize plaque characterization in terms of its chemical and morphologic characteristic. The new tool can lead to the development of new methods of interpreting the newly obtained data. In this study, the algorithm to fully automated lipid pool detection on NIRS images is proposed.* Method*. Designed algorithm is divided into four stages: preprocessing (image enhancement), segmentation of artifacts, detection of lipid areas, and calculation of Lipid Core Burden Index.* Results*. A total of 31 NIRS chemograms were analyzed by two methods. The metrics, total LCBI, maximal LCBI in 4 mm blocks, and maximal LCBI in 2 mm blocks, were calculated to compare presented algorithm with commercial available system. Both intraclass correlation (ICC) and Bland-Altman plots showed good agreement and correlation between used methods.* Conclusions*. Proposed algorithm is fully automated lipid pool detection on near infrared spectroscopy images. It is a tool developed for offline data analysis, which could be easily augmented for newer functions and projects.

## 1. Introduction

### 1.1. Clinical Importance and Motivation

Data from autopsy studies showed that plaque rupture of a coronary artery is a leading cause of myocardial infarction [1]. Identification of atherosclerotic plaques prone to rupture, commonly known as vulnerable plaques, is still a challenge for modern cardiology. Vulnerable plaques characterized as “positive vessel remodeling” have a lipid core and a fibrous cap less than 65 *μ*m thick. These features define them as a thin-cap fibroatheroma (TCFA) [2–5]. If a TCFA ruptures, it exposes the plaque's core to platelets, and the thrombosis of coronary arteries occurs [6]. Previous* in vivo* observations showed that the presence of TCFA is an independent risk factor of major adverse cardiac events (MACE) [7].

**Figure 1:**
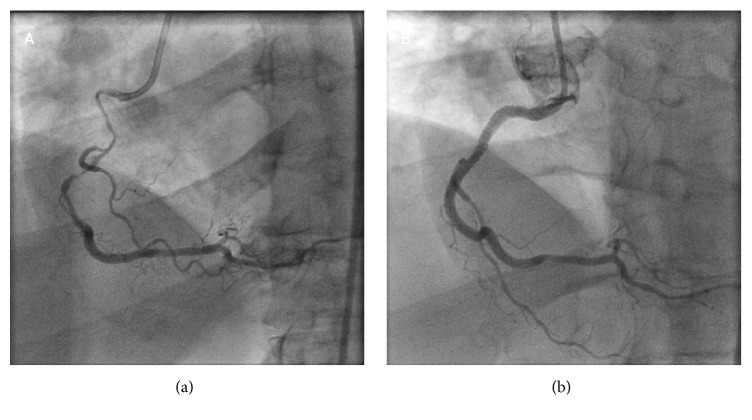
(a) shows the angiographic image of right coronary artery before stenting with visible stenosis in proximal and mid segment. (b) shows the same vessel after stenting.

Conventional angiography is limited regarding the identification of lipid core plaque (LCP) [8] because it only presents vessel's lumen without insight into the vessel's wall (Figure 1). It leads to the underestimation of the atherosclerosis burden, as the positive vessel remodeling allows for the maintenance of “normal” lumen size, despite the considerable plaque.

**Figure 2:**
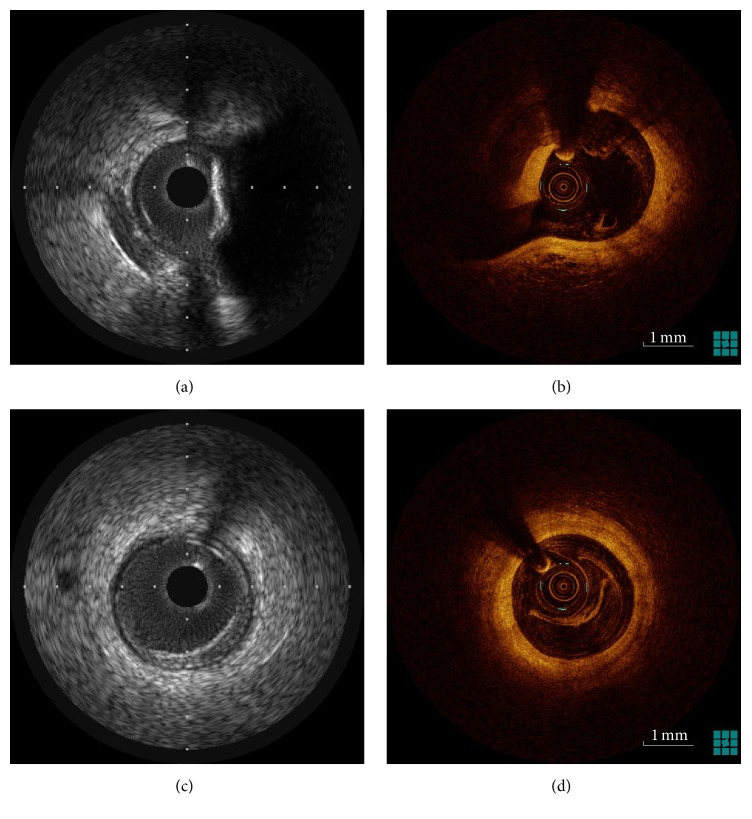
(a) shows a cross-sectional IVUS image with visible calcification and bifurcation. (b) shows the same location but now acquired by OCT. (c) and (d) show the normal vessel in IVUS and OCT, respectively.

These limitations may be overcome by the use of intracoronary imaging (Figure 2): intravascular ultrasound (IVUS) [7, 9] and optical coherence tomography (OCT) [10, 11]. They provide quantitative and qualitative analysis of the plaque, detect plaque rupture, present thrombosis, and guide PCI [12]. IVUS presents the plaque burden and vessel remodeling but is limited regarding the analysis of tissue composition. On the other hand, OCT describes the chemical composition of the plaque and detects TCFA, but, due to its limited penetration into the vessel wall, the assessment of plaque burden and vessel's remodeling is hampered.

During the recent years, we witnessed a sort of competition at the field of MACE risk stratification between available intravascular imaging but still a final word has not been said.

### 1.2. State of the Art

Intracoronary imaging techniques have been intensively developed over the last two decades to get the perfect tool. The ideal imaging modality should be able to obtain information about the plaque burden, vessel remodeling, and lumen stenosis and should detect calcifications, lipids, plaque rupture, and TCFA and help optimize PCI results. Recently a novel intravascular imaging modality has been introduced, which makes us closer to receive the universal imaging tool. It is a fusion of two techniques: IVUS and near infrared spectroscopy (NIRS) (TVC imaging system, Infraredx company). NIRS alone is oriented to the detection of lipids within the vessel wall but coregistered with IVUS images provides information about the plaque composition and its burden simultaneously. As it was previously presented, NIRS-IVUS poses the ability to detect vulnerable plaques [1, 2, 13–16].

With the advent of such a new intravascular imaging modality, there is still a need for new methodologies, computer-assisted quantitative and qualitative tools to evaluate and interpret the obtained data. Such tools should help in the real time data acquisition and assessment, which may be achieved by its integration with the IT systems of catheterization laboratories. In addition, such tools should also help in offline data analysis and should allow for more demanding analysis.

The purpose of this study is to demonstrate a new fully automated estimation of lipid burden by the analysis of data from NIRS. Such a new tool should be enriched with new functions and be universal regarding NIRS data analysis.

This paper is organized in 5 sections as follows: Section 1 presents the motivation of our work and the review of the state of the art in the area of NIRS's images.  Section 2 specifies the overview of the implemented algorithm, including chemogram preprocessing, artifact detection, lipids core plaque segmentation, and calculation of basic features. In Sections 3 and 4, the conducted statistical analysis tests and results are described.  Section 5 closes the paper with conclusions and highlights future directions.

## 2. Methods and Materials

**Figure 3:**
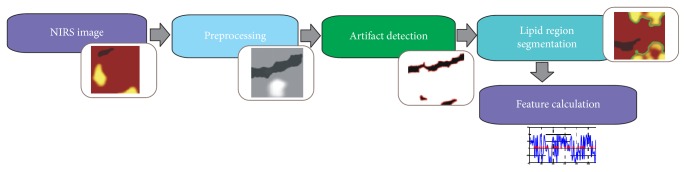
Proposed method for the lipid core plaque detection on NIRS images.

We would like to present our algorithmic solutions to solve the problem of lipid core plaque detection on NIRS images. The first part presents in detail the technical and medical aspects of chemograms. Images used in this study were acquired via TVC Imaging System*™* and TVC Insight*™* Catheters, from 31 patients. In the second part, we concentrate on the implemented diagnostic system with its particular steps. The flowchart of the proposed system is illustrated in Figure 3. The implemented application is divided into four stages: preprocessing (image enhancement), segmentation of artifacts, detection of lipid areas, and calculation of medically important features.

### 2.1. NIRS Images

**Figure 4:**
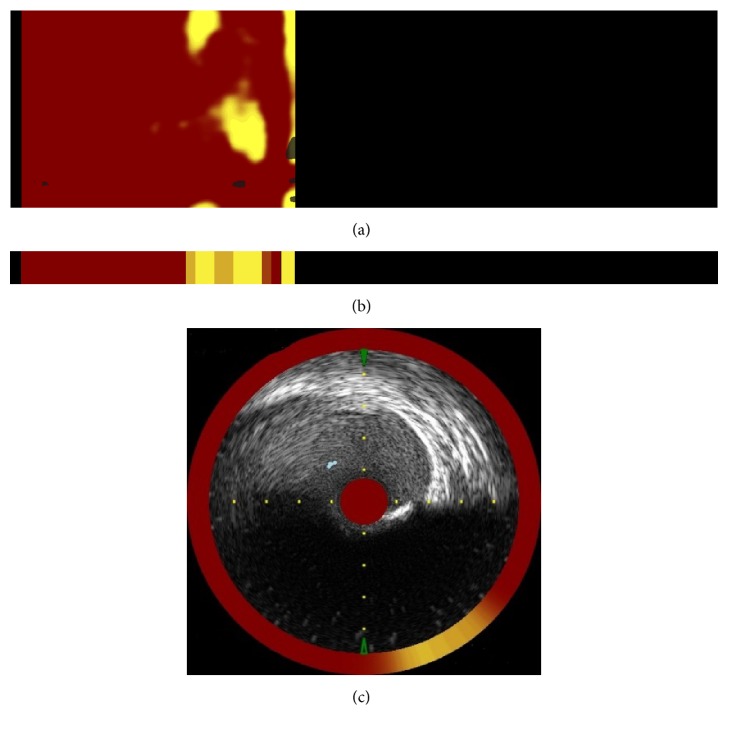
(a) shows exemplary chemogram and (b) shows corresponding block chemogram. (c) shows cross-sectional IVUS image with corresponding NIRS data.

The TVC Imaging System (Infraredx) is the intravascular imaging system with the ability to assess vessel composition and its structure. The system is composed of a mobile console with an automated pullback and rotation device and dual-modality TVC Insight Catheter for simultaneous, coregistered acquisition of NIRS lipid core plaque (LCP) and grayscale IVUS. By obtaining views of plaque composition in terms of both its chemical (by NIRS) and its morphologic (by IVUS) characteristics [17], scanning with automated rotational pullback is performed at a speed of 0.5 mm/s. The system performs approximately 32,000 chemical measurements per 100 mm of artery scanned. After near infrared light emission, a detector measures the amount of near infrared light reflected at different wavelengths to determine tissue composition [17]. A predictive algorithm calculates the probability that LCP is present at each interrogated location in the artery and, after pullback, NIRS lipid core data are immediately and automatically displayed in a two-dimensional map of the vessel called a “chemogram” (Figure 4).

The *x*-axis of the chemogram represents mm of pullback in the artery and the *y*-axis represents degrees of rotation; a colour scale from red to yellow indicates increases of lipids. The “block chemograms” (Figure 4) provides a semiquantitative summary metric of the results for each 2 mm section of artery. The numerical value of each block in the block chemogram represents the 90th percentile of all pixel values in the corresponding 2 mm chemogram segment. The block chemograms are mapped to the same colour scale as the chemogram, and the display is grouped into four discrete colours to aid in visually interpreting the algorithm probability that a LCP is present in that 2 mm block (red: *p* < 0.57; orange: 0.57 ≤ *p* ≤ 0.84; tan: 0.84 ≤ *p* ≤ 0.98; yellow: *p* > 0.98) [17]. Additionally, the NIRS data is mapped and paired with corresponding IVUS frames, as a ring around the IVUS image (Figure 4).

While the chemogram helps in visual interpretation, the Lipid Core Burden Index (LCBI) is a semiquantitative summary metric of the total LCP, detected in the scanned segment, and it is computed as the fraction of valid pixels within the scanned region that exceed a LCP probability of 0.6 multiplied by 1,000 [17].

The accuracy of the NIRS system in detecting LCPs was confirmed in an autopsy study, where the block chemogram produced by NIRS was compared with histology of 2 mm segments (AUC = 0.80, 95% CI: 0.77–0.97) [8, 14].

### 2.2. NIRS Image Preprocessing

**Figure 5:**
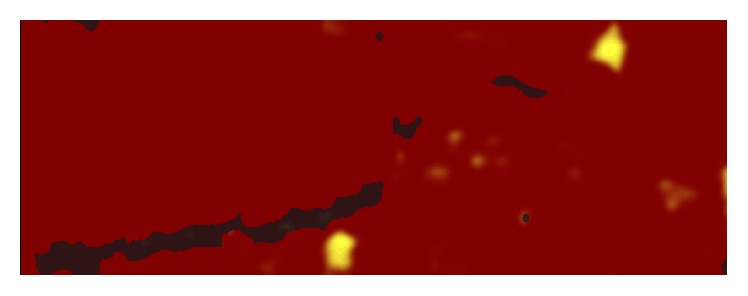
Chemogram of a stented area with visible artifacts (dark areas) and lipid core plaque (yellow areas).

The main goal of the preprocessing step is to improve the image quality by reducing or even removing the unrelated and/or surplus parts in the NIRS images [18]. In Figure 5, we present an exemplary chemogram, where red pixels indicate the arterial wall and yellow pixels the presence of lipid regions and black regions (artifacts) and the presence of calcifications or the guide wire. If a pixel does not contain enough data, it appears black [19], where a contiguous black region could be caused by guide-wire shadowing. Also, the ability of near infrared light to penetrate through calcium could cause “shadowing” on lipid pools, which are covered by calcium [14]. In cases where most of chemograms include black regions, it could be easily suggested that there was a problem with NIRS-IVUS probe during signal acquisition.

**Figure 6:**
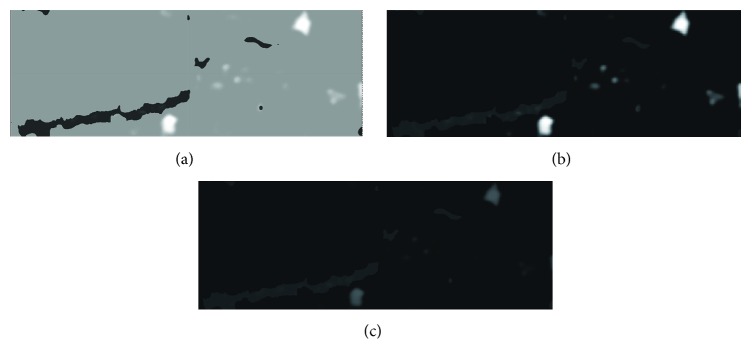
RGB channels converted to grayscale for NIRS image: (a) red channel, where artifacts become clear and visible, (b) green channel, where lipid areas are more perceptible, and (c) blue channel.

The aim of this research is to detect not only the lipid regions, which are clearly visible, but also those hidden under artifacts. To achieve this, we propose to preprocess and analyze each of the RGB image channels. An RGB image, which is 24 bits, has three 8-bit channels: red, green, and blue.  Figure 6 presents particular channels after converting them to grayscale for the chemogram presented in Figure 5.

The analysis of the red channel gives the opportunity to detect the artifacts very precisely, while the processing of the green channel allows detection of lipid structures, which are not covered by the artifacts. The preprocessing step for each of the channels includes an image intensity adjustment. This step maps the intensity values in grayscale image I to new values in J, such that 1% of data is saturated at low and high intensities of I. This increases the contrast of the output image J.

### 2.3. Detection of Artifacts

**Figure 7:**
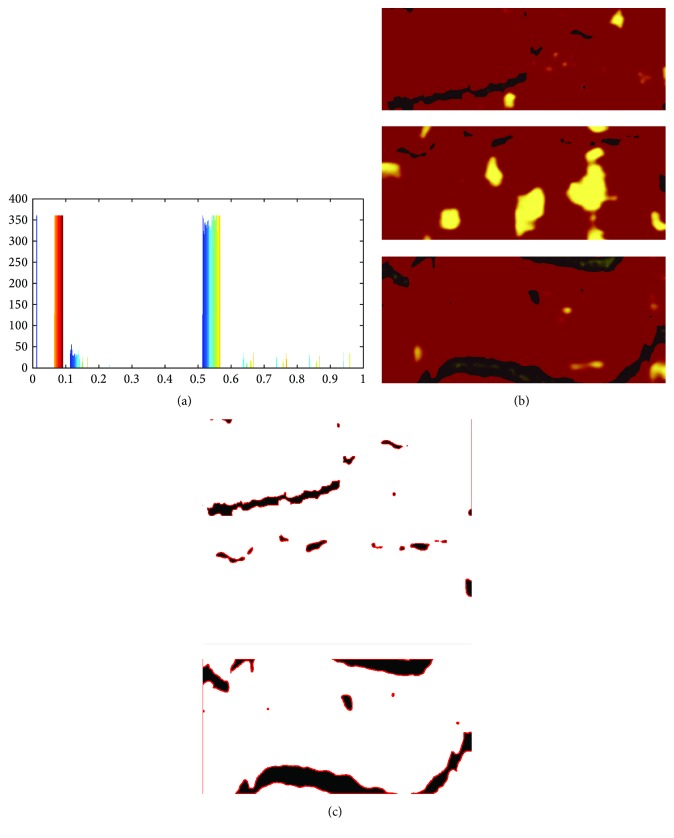
Detection of artifacts in the NIRS images. (a) Histogram which serves to show the sense of applying the Otsu algorithm. (b) Original NIRS images. (c) Marked artifacts' areas.

The segmentation of artifacts is the next step of the proposed algorithm. The detection of these regions is important in terms of segmenting lipid regions that lie under the artifacts. Segmentation of specific regions within an image involves separating an image into regions (or their contours) corresponding to different objects. The regions are segmented by identifying common properties or contours by distinguishing differences between regions (edges). The simplest property that pixels in a region can share is intensity. Therefore, a natural way to segment such regions is through thresholding, the separation of light and dark regions. Seeing that the intensity difference between the artifacts and the background as well as lipid regions is high enough, we propose to use the Otsu thresholding method. Otsu's method, named after Nobuyuki Otsu, is used to automatically perform clustering-based image thresholding, which means the reduction of a gray level image to a binary image. Thresholding is one of the widely used methods for image segmentation. It is useful in discriminating foreground from the background. By selecting an adequate threshold value *T*, the gray level image can be converted to binary image. The algorithm assumes that the image contains two classes of pixels following bimodal histogram (foreground pixels and background pixels). It then calculates the optimum threshold separating the two classes so that their combined spread (intraclass variance) is minimal or equivalent (because the sum of pair-wise squared distances is constant), so that their interclass variance is maximal [20]. This step is performed on images gained from the red channel after the intensity adjustment step.  Figure 7 shows an exemplary histogram and several results of the segmented areas.

### 2.4. Segmentation of Lipid Core Plaque

**Figure 8:**
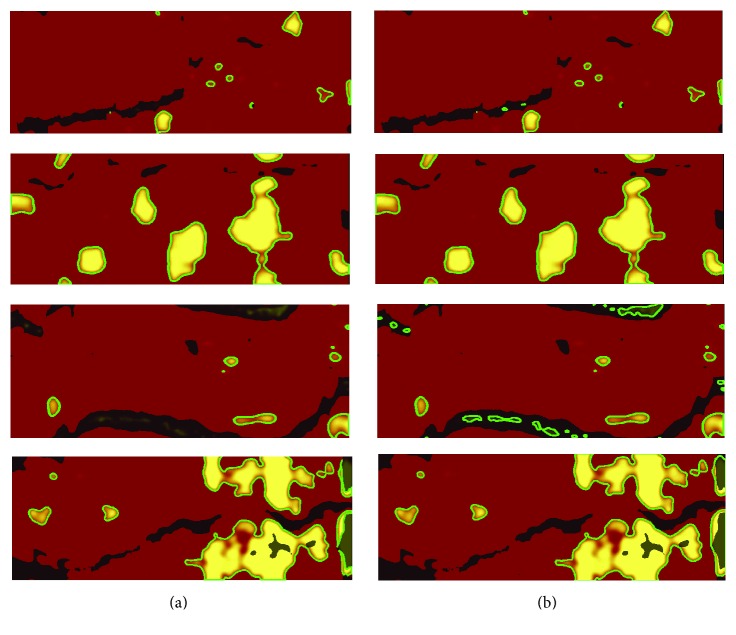
Segmentation of the lipid core plaque areas: (a) detection of the visible regions and (b) detection of lipid regions covered with artifacts.

The detection of lipid regions consists of two steps including segmentation of visible regions and those lying under the artifacts. For the detection of clearly visible regions, we use the Otsu algorithm described in previous section. The outcome of this step can be seen in Figure 8(a). The detection of lipid regions hidden under artifacts is performed by the analysis of the intensity changes in these regions. Firstly, we compute a histogram for the segmented regions. The highest peak informs us about the intensity of the analyzed region (background of the artifact region). Secondly, based on the value of the background we study the whole region looking for intensity changes. If the difference between the background intensity is higher than the experimental threshold, the region is marked as a lipid area (Figure 8(b)).

Figure 8 presents the outlined segmented areas of the lipid core plaques.

### 2.5. Calculation of Medically Important Features

From the clinical point of view in our study, the most important parts of the chemogram are those that lie in the stented segment. During the automatic analysis of the NIRS image, the physician can determine the region of interest. LCBI is helpful in assessing the risk of plaque rupture and the use of preventative strategies during PCI. Plaque with large LCP and identified by NIRS maxLCBI_4 mm_ of ≥500 suggested high risk plaque [2, 15, 21].

Within the region, the following parameters are calculated:

LCBI_total_: pixels assigned to the lipid plaque regions were divided by all viable pixels to generate the Lipid Core Burden Index,

(1) $$\begin{array}{c} \text{LCB}{\text{I}}_{\text{total}}=\frac{\text{Area}\left(\text{Lipid Core Plaque}\right)}{\text{Area}\left(\text{Stented/Selected}\right)}*1000. \end{array}$$

 maxLCBI_2 mm_ is the maximal LCBI in any 2 mm-long segment.
 maxLCBI_4 mm_ is the maximal LCBI in any 4 mm-long segment.

## 3. Statistical Analysis

Continuous parameters were reported as a median with the first and the third quartiles (Q1: 25%, Q3: 75%). The Wilcoxon signed-rank test (paired Wilcoxon test) was used for comparison between two related samples. In cases where *p* value is greater than 0.05, test informed us that there is no reason to treat measurements as significantly different, but it is still not enough to prove that outcomes from our method are similar to the second method. To check if the measurements from both methods are similar, the intraclass correlation (ICC) was computed. The higher the ICC, the higher the relation between lipid detection methods. Additionally, Bland-Altman plots were presented. Analyses were performed in R, language and environment for statistical computing (R Core Team 2014, Vienna, Austria).

## 4. Results and Discussion

For the validation of the proposed fully automated detection of lipid regions method, we used NIRS images acquired from 31 patients. The data were provided by the Medical University of Silesia.

### 4.1. Database Specification

In order to validate the proposed method, we compared the two methods: method 1, commercial available system; method 2, our algorithm. 31 chemograms were analyzed in terms of lipid pool detection and automated calculations of LCBI total (maximum LCBI in 4 mm blocks and maximum LCBI in 2 mm blocks) in stented segment.

### 4.2. Validation of Automated Lipid Detection

**Figure 9:**
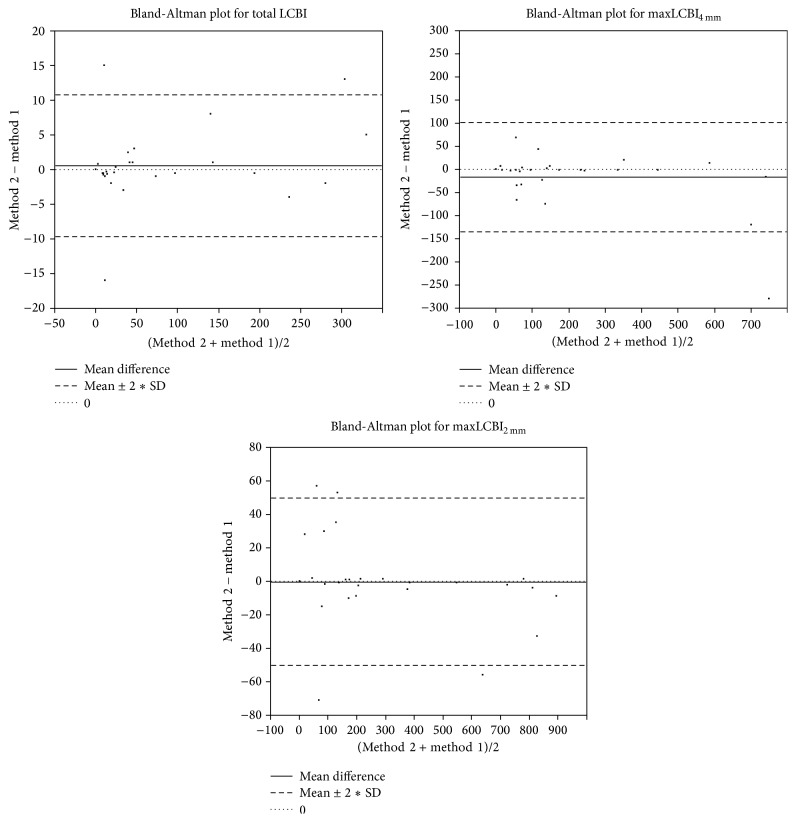
Bland-Altman plot for total LCBI, maxLCBI_4 mm_, and maxLCBI_2 mm_ calculated by two methods.

The obtained results are collected in Table 1. For all measured parameters, total LCBI, maxLCBI_2 mm_, and maxLCBI_4 mm_, the ICC is very close to value of 1 showing that the parameters calculated by our method (method 2) are similar to obtained results from commercially available system. Also, the Bland-Altman plots (Figure 9) indicate a good agreement between used methods. Most points plotted are between the solid line (mean diff.) and the dashed line (mean ± 2*∗*standard  deviation).

### 4.3. Discussion

In this paper, a fully automated detection of lipid method, for detecting lipid pools and automated calculation of LCBI in NIRS images, is presented. The method detects automatically the lipid region border even if they are covered by “a shadowing” artifact. Also, there is possibility to calculate LCBI in chosen region and maximal value of LCBI in different size of blocks to better match assessed region with IVUS data. Additionally, our algorithm has a tool to automated detection of maximal lipid arc in an assessed block.

We noticed that our algorithm has 100% lipid detection; maximum LCBI in 4 mm and in 2 mm blocks are different between our method and method presented by commercially available system. Taking into account that *p* value calculated by the Wilcoxon signed-rank test is insignificantly different, the *p* value is not as high as that in total LCBI. Probably, it is a result of the region being divided into blocks of 4 mm and not analyzed while moving a window with a width of 4 mm.

## 5. Conclusion and Future Plans

Characterization of the atherosclerotic plaque and detecting and identifying lipid core are very important for diagnosing and treating coronary artery disease. Development of a NIRS-IVUS tool to detect lipid-rich plaque confirmed that lipid-rich plaques play a major role in stenting complications and adverse events. Nevertheless, there is still the need to develop better and more accurate methods, tools which will be easily available not only for clinicians in cath labs but also for researchers and investigators, to obtain more accurate information from intracoronary images. Next steps in furthering our study will be improving our algorithm for a more precise calculation of maximal LCBI. The development of functions for 3D vessel reconstruction, based on OCT and IVUS, with a wrapped NIRS chemogram, will enable better visualization of lipid localization on the vessel wall.

## Figures and Tables

**Table 1:** Statistical comparison of parameters.

	Method 1	Method 2	Difference	Relative difference	Paired Wilcoxon *p* value	ICC (95% CI)	ICC *p* value
Total LCBI	23.00 (9.00–97.00)	22.60 (8.40–96.50)	0.00 (−0.80–1.00)	−0.61% (−6.17%–2.83%)	0.8822	1 (1-1)	<0.0001
MaxLCBI_4 mm_	95.00 (20.00–245.00)	94.20 (23.00–242.00)	−1.50 (−16.00–1.20)	−1.55% (−16.19%–2.81%)	0.1265	0.97 (0.93–0.98)	<0.0001
MaxLCBI_2 mm_	162.00 (43.00–385.00)	163.10 (45.00–384.00)	0.00 (−5.00–1.20)	−0.28% (−2.46%–1.62%)	0.4645	1 (0.99–1)	<0.0001
